# NAD^+^ Precursors Repair Mitochondrial Function in Diabetes and Prevent Experimental Diabetic Neuropathy

**DOI:** 10.3390/ijms23094887

**Published:** 2022-04-28

**Authors:** Krish Chandrasekaran, Neda Najimi, Avinash R. Sagi, Sushuma Yarlagadda, Mohammad Salimian, Muhammed Ikbal Arvas, Ahmad F. Hedayat, Yanni Kevas, Anand Kadakia, James W. Russell

**Affiliations:** 1 Department of Neurology, University of Maryland School of Medicine, Baltimore, MD 21201, USA; kchandrasekaran@som.umaryland.edu (K.C.); neda_najimi@yahoo.com (N.N.); avinashrao.sagi@gmail.com (A.R.S.); ysushuma@gmail.com (S.Y.); mohammad.salimian@gmail.com (M.S.); m.ikbalarvas@gmail.com (M.I.A.); fahim.hedayat@yahoo.com (A.F.H.); ykevas@terpmail.umd.edu (Y.K.); akadakia1991@gmail.com (A.K.); 2 Veterans Affairs Medical Center, Baltimore, MD 21201, USA; 3 CAMC Institute for Academic Medicine, 415 Morris Street Suite 300, Charleston, WV 25301, USA

**Keywords:** sirtuins, diabetic neuropathy, NAD^+^, mitochondria, NEDD4-1

## Abstract

Axon degeneration in diabetic peripheral neuropathy (DPN) is associated with impaired NAD^+^ metabolism. We tested whether the administration of NAD^+^ precursors, nicotinamide mononucleotide (NMN) or nicotinamide riboside (NR), prevents DPN in models of Type 1 and Type 2 diabetes. NMN was administered to streptozotocin (STZ)-induced diabetic rats and STZ-induced diabetic mice by intraperitoneal injection at 50 or 100 mg/kg on alternate days for 2 months. mice The were fed with a high fat diet (HFD) for 2 months with or without added NR at 150 or 300 mg/kg for 2 months. The administration of NMN to STZ-induced diabetic rats or mice or dietary addition of NR to HFD-fed mice improved sensory function, normalized sciatic and tail nerve conduction velocities, and prevented loss of intraepidermal nerve fibers in skin samples from the hind-paw. In adult dorsal root ganglion (DRG) neurons isolated from HFD-fed mice, there was a decrease in NAD^+^ levels and mitochondrial maximum reserve capacity. These impairments were normalized in isolated DRG neurons from NR-treated mice. The results indicate that the correction of NAD^+^ depletion in DRG may be sufficient to prevent DPN but does not significantly affect glucose tolerance, insulin levels, or insulin resistance.

## 1. Introduction

Diabetic peripheral neuropathy (DPN) is a common neurological complication of diabetes. Currently, there is no therapy that permanently prevents or reverses neuropathy. Strict glycemic control ameliorates but does not reverse neuropathy in type 1 diabetes (T1D) patients and is relatively ineffective at preventing diabetic neuropathy in type 2 diabetes (T2D) [1]. There is emerging evidence that lifestyle interventions are effective in individuals with established diabetic neuropathy. In addition, there is evidence that effects on biochemical pathways that improve muscle function affect other organ systems, including peripheral nerves. These data are reviewed in [2]. In addition to hyperglycemia, dyslipidemia has been suggested as a major contributor to DPN in T2D (reviewed in [3,4,5]). Defective mitochondrial (Mt) fatty acid oxidation may lead to the accumulation of the precursor fatty acylcarnitine in neurons and subsequent neuronal Mt dysfunction leading to axonal degeneration and neuropathy [6,7,8,9,10]. As a result of Mt damage, oxidative stress occurs in dorsal root ganglion (DRG) neurons, axons, and Schwann cells, and has been proposed as a unifying mechanism for diabetic neuropathy [9,11,12,13,14]. Neurons have a large surface area and demand high energy to maintain their membrane potential [15]. The survival of distal axons, which in humans may be a considerable distance from the soma, is dependent on axonal transport mechanisms that also require energy. The net effect of Mt dysfunction is that it results in a low aerobic capacity to meet the energy demand within the neuron. Over time, this can lead to neuronal and axonal degeneration.

NAD^+^ is critical in maintaining Mt function, and NAD^+^ levels decline with age in individuals with neurodegenerative conditions, acute brain injury, obesity, or diabetes [11,16,17,18]. Several studies have shown that the loss of NAD^+^ leads to axonal degeneration in peripheral neuropathy [19,20,21]. NAD^+^ is used as a degradation substrate for enzymes such as sirtuins, poly (ADP-ribosyl) transferase 1 (PARP1), and cluster of differentiation 38 (CD38) [18,22,23]. Axonal degeneration can be promoted by the activation of sterile alpha and TIR motif constraining 1 (SARM1) or deleted in bladder cancer protein 1 (DBC1), which inhibits Sirtuin 1 (SIRT1) activity [22,23,24]. The activation of PARP1 or CD 38 also promotes axonal degeneration, whereas the knockout of PARP1, CD38, SARM1, and DBC1 protect mice against high fat diet (HFD)-induced neuropathy or chemotherapy-induced neuropathy [24,25,26,27]. Furthermore, proteins that resynthesize NAD^+^ via the salvage pathway, such as NMNAT1–3, protect against axonal degeneration [28,29,30,31,32]. Among the NAD^+^ consuming enzymes, SIRT1 is unique because its activation protects against DPN, whereas the inhibition of other NAD^+^ consuming proteins protects against DPN [11,12,33].

Nicotinamide riboside (NR) may improve diabetes and diabetic neuropathy in T2D [19]. However, it is unknown whether nicotinamide mononucleotide (NMN) can prevent experimental diabetic neuropathy and the NR-regulated mechanisms that prevent neuropathy. In this study, we tested whether the administration of the NAD^+^ precursors NMN or NR would protect against streptozotocin (STZ) or high fat diet (HFD)-induced peripheral neuropathy. We hypothesize that NMN and NR, by increasing NAD^+^ levels in DRG neurons, can furnish reducing equivalents (NADH) to the mitochondrial electron transport chain to generate ATP, deacetylating proteins—including mitochondrial proteins to regulate fatty acid oxidation—and thereby preventing diabetic neuropathy.

## 2. Results

### 2.1. Administration of NMN Prevents STZ-Induced Increases in Triglyceride and Non-Esterified Fatty Lipid Levels in Rat Blood

Induction of T1D with STZ in Sprague Dawley (SD) rats caused significant changes in the following blood parameters that were not protected by NMN administration: decrease in body weight from 450 ± 17 g to 346 ± 14 g (*p* < 0.001, Table 1); increase in blood glucose levels from 132 ± 18 mg/dL to 405 ± 22 mg/dL (*p* < 0.001, Table 1); increase in % HbA1c from 5.4 ± 1 to 16.7 ± 4.1 (*p* < 0.001, Table 1); decrease in insulin levels from 8.3 ± 2 to 1.2 ± 0.7 (*p* < 0.001, Table 1); and increase in total cholesterol, HDL, and LDL levels. A comparison of the changes in body weight from baseline to the end of the study showed that administration of STZ, irrespective of NMN treatment, caused a significant decrease in body weight. The administration of NMN significantly decreased STZ-induced increases in the following: the STZ-induced increase in triglycerides was decreased from 493 ± 23 mg/dL to 361 ± 19 mg/dL (*p* < 0.05, Table 1) and non-esterified fatty acids (NEFA) from 6.6 ± 1.8 mM to 3.8 ± 1 (*p* < 0.05, Table 1). The measurement of the intraperitoneal glucose tolerance test (IP-GTT; *n* = 6) showed a significant increase in the area under the curve (AUC) in STZ rats compared to non-diabetic rats, but there was no significant difference in AUC between STZ and STZ + NMN rats (Supplementary Figure S1 in reference [16]).

### 2.2. Administration of NMN Prevents STZ-Induced Peripheral Neuropathy in Rats

**Figure 1:**
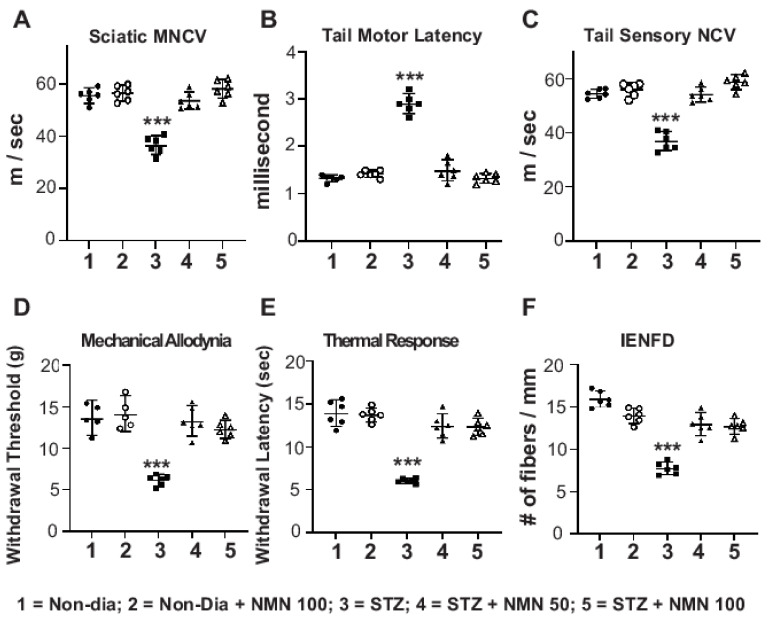
NMN prevents STZ-induced neuropathy in SD rats (*n* = 6/group). Thirty male SD rats were randomly assigned to five groups of six rats per group. Group # 1: non-diabetic (non-Dia); Group # 2: non-diabetic with NMN (non-Dia + NMN) (100 mg/kg); Group # 3: streptozotocin (STZ); Group # 4: streptozotocin with NMN (STZ + NMN) (50 mg/kg); and Group # 5: streptozotocin with NMN (STZ + NMN) (100 mg/kg). Injections (IP) of saline and NMN were administered on alternate days for 2 months. Rats were tested for the following parameters: SMNCV (**A**), TML (**B**), TSNCV (**C**), mechanical allodynia by von Frey filament paw withdrawal threshold (**D**), paw thermal sensitivity by Hargreaves test (**E**), and IENFD of the hind paw (**F**). Statistical comparisons were made between the five groups by ANOVA and post hoc Tukey test. *** *p* < 0.001; STZ, Group # 3 at 2 months compared to all other groups in all parameters.

We tested whether NMN injection could prevent peripheral neuropathy in STZ rats. After 8 weeks of STZ-induced diabetes, rats showed a significant slowing of SMNCV (Figure 1) (from 55.61 ± 2.8 m/s in non-diabetic to 36.6 ± 3.4 in STZ; *p* < 0.001), increased TML latency (from 1.33 ± 0.07 ms to 2.9 ± 0.2 ms; *p* < 0.001), and decreased TSNCV (from 54.3 ± 1.6 m/s in non-diabetic to 36.9 ± 3.4 m/s in STZ rats; *p* < 0.001). These changes in nerve conduction velocity (NCV) in STZ rats were consistent with the development of peripheral neuropathy. After 8 weeks, there was a significant decrease in the von Frey paw withdrawal threshold in STZ compared to non-diabetic mice (non-Día) = 14 ± 1 g vs. STZ = 6.2 ± 0.7 g; *p* < 0.001), consistent with the development of tactile allodynia. Likewise, there was a decrease in thermal sensitivity in the Hargreaves test from 14 ± 1.5 s in non-diabetic rats to 6 ± 0.3 s in STZ rats. In contrast, the administration of NMN at both 50 mg/kg and 100 mg/kg preserved NCVs. NCVs were comparable in non-diabetic rats with or without NMN. For example, the SMNCVs were similar (*p* = 0.85 between non-diabetic vs. STZ + NMN 50 mg/kg or *p* = 0.88 between non-diabetic + NMN 100 mg/kg vs. STZ + NMN 100 mg/kg, Figure 1). The administration of NMN to STZ rats similarly preserved STZ-induced changes in TML and SNCV. STZ + NMN rats had normal tactile allodynia at 8 weeks while STZ rats demonstrated a decreased von Frey paw withdrawal threshold (Figure 1).

Eight weeks after injecting both non-diabetic and STZ rats with NMN, the rats were euthanized and their paw skins were examined for IENFD (Figure 1F). Skin biopsies showed a significant decrease in the IENFD of STZ rats (7.7 ± 0.7 fibers/mm) compared to non-diabetic rats (15.9 ± 0.9 fibers/mm; *p* < 0.001). There was no significant change in the IENFD (15.9 ± 0.9 fibers/mm to 14 ± 0.9 fibers/mm; *p* = 0.65) in non-diabetic rats compared to non-diabetic + NMN rats. In STZ + NMN rats, there was no significant change in the IENFD compared to non-diabetic + NMN rats (STZ + NMN 50 = 13 ± 1.3 vs. non-diabetic + NMN 100 = 14 ± 0.9 fibers/mm, *p* = 0.41: STZ + NMN 100 = 12.7 ± 0.9 vs. non-diabetic + NMN 100 = 14 ± 0.9 fibers/mm, *p* = 0.2). In contrast, the IENFD count was still higher in STZ + NMN rats compared to STZ rats (STZ = 7.7 ± 0.7 fibers/mm vs. STZ + NMN 50 = 13 ± 1.3; *p* < 0.001; vs. STZ + NMN 100 = 12.7 ± 0.9; *p* < 0.001; STZ + NMN 50 vs. STZ + NMN 100; *p* = 0.98).

### 2.3. Administration of NMN Prevents STZ-Induced Peripheral Neuropathy in Mice

We next determined whether NMN could prevent neuropathy in STZ diabetic mice. The blood and nerve conduction results are shown in Table 2. There was a significant decrease in body weight; increase in blood glucose levels from 105 ± 12 mg/dL to 405 ± 22 mg/dL (*p* < 0.001, Table 2); increase in % HbA1c from 5.4 ± 1 to 15.7 ± 3.1 (*p* < 0.001, Table 2), decrease in insulin levels from 0.8 ± 0.2 to 0.2 ± 0.05 (*p* < 0.001, Table 2); and increase in total cholesterol, HDL, and LDL levels. In contrast, the administration of NMN significantly decreased the STZ-induced increase in triglycerides from 93 ± 13 mg/dL to 61 ± 10 mg/dL (*p* < 0.05, Table 2) and NEFA from 6.4 ± 1.4 mM to 3.6 ± 0.8 (*p* < 0.05, Table 2). Measurements of the IP-GTT (*n* = 6) showed a significant increase in the area under the curve (AUC) in STZ mice compared to non-diabetic mice, but there was no significant difference in the AUC between STZ and STZ + NMN mice (Supplementary Figure S1).

After 8 weeks of STZ-induced diabetes, C57Bl6 mice showed a significant slowing of SMNCV (from 55 ± 7 m/s in non-diabetic to 26 ± 6 in STZ; *p* < 0.001), decreased TMNCV (from 45 ± 4 m/s to 24 ± 3 m/s; *p* < 0.001), decreased TSNCV (from 41 ± 3 m/s to 27 ± 5 m/s; *p* < 0.001), the development of mechanical allodynia, and decreased thermal sensitivity of hind paws (Table 2). The administration of NMN at both 50 mg/kg and 100 mg/kg ameliorated these STZ-induced changes in nerve conduction parameters (Table 2). The administration of NMN to STZ mice showed normal tactile allodynia at 8 weeks, while the von Frey paw withdrawal threshold was decreased in STZ mice (Table 2). Hind paw skin biopsies showed a significant decrease in the IENFD in STZ mice compared to non-diabetic mice. There was no significant change in the IENFD in non-diabetic mice compared to non-diabetic + NMN mice. In contrast, the IENFD was higher in STZ + NMN mice compared to STZ mice (STZ = 12 ± 2 fibers/mm vs. STZ + NMN 50 = 23 ± 3; *p* < 0.001; vs. STZ + NMN 100 = 24 ± 3; *p* < 0.001). These results suggest that IP administration of NMN protected against T1D-induced peripheral neuropathy.

### 2.4. Dietary Administration of NR Prevents HFD-Induced Increases in Triglyceride and NEFA Levels in Mice

Several groups, including ours, have shown that feeding C57Bl6 mice with HFD induces peripheral neuropathy. We tested whether the neuropathic changes could be ameliorated by the dietary administration of NR to HFD-fed mice. The dietary addition of NR did not alter HFD-induced weight gain or increases in plasma blood glucose, Hb1Ac, insulin, or cholesterol (HDL and LDL) levels but did alter other lipid levels (Group 3 vs. Groups 4 and 5 in Table 3). In the HFD + NR mice (*n* = 8), there was a decrease in triglyceride (HFD = 93 ± 23 mg/dl vs. HFD + NR 150 mg = 76 ± 12 mg/dl or HFD + NR 300 mg = 61 ± 10 mg/dl; *p* < 0.05) and NEFA (HFD = 6.6 ± 1.8 mM vs. HFD + NR 150 mg = 3.6 ± 0.7 mM; *p* < 0.05 or HFD + NR 300 mg = 4.8 ± 1 mM) levels. The IP-GTT (*n* = 6) showed a significant increase in the AUC in HFD mice compared to CD mice but there was no significant difference in the AUC between HFD and HFD + NR 150 or 300 mg/kg mice or between CD and CD + NR 300 mg/kg mice (Table 3 and Supplementary Figure S2).

### 2.5. Dietary Administration of NR Prevents Peripheral Neuropathy Induced by an HFD

**Figure 2:**
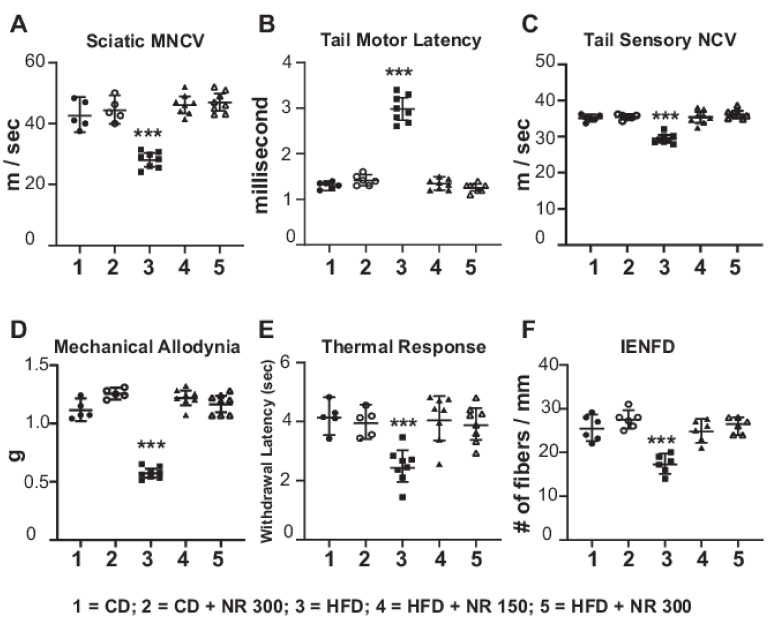
NR prevents HFD-induced neuropathy in C57Bl6 mice (*n* = 6 to 8 per group). WT C57BL6 mice were randomly assigned to five groups of six to eight mice per group. Group #1: CD; Group # 2: CD + NR 300 mg/kg; Group # 3: HFD; Group # 4: HFD + NR (150 mg/kg); and Group # 5: HFD + NR (300 mg/kg). NR was added to the diet at the dose mentioned to the groups indicated for 2 months. The mice were tested for the following parameters: sciatic motor nerve conduction velocity, SMNCV, (**A**), tail motor latency, TML, (**B**), tail sensory nerve conduction velocity, TSNCV, (**C**), mechanical allodynia by von Frey filament paw withdrawal threshold (**D**), paw thermal sensitivity by Hargreaves test (**E**), and IENFD of the hind paw (**F**). Statistical comparisons were made between the five groups by ANOVA and post hoc Tukey test. *** *p* < 0.001; HFD, Group # 3 at 2 months compared to all other groups in all parameters.

We tested whether dietary administration of NR could prevent HFD-induced peripheral neuropathy. After 8 weeks of HFD feeding (*n* = 8), C57BL6 mice showed a significant slowing of SMNCV (Figure 2) (from 42.77 ± 2.09 m/s in CD to 29.88 ± 1.23 m/s in HFD; *p* < 0.001), increased TML (from 1.3 ± 0.07 ms to 2.89 ± 0.3 ms; *p* < 0.001), and decreased TSNCV (from 35.1 ± 0.9 m/s in CD to 29.34 ± 1.34 m/s in HFD; *p* < 0.001). These changes in NCV in HFD-fed mice were consistent with the development of peripheral neuropathy. After 8 weeks of HFD, there was a significant decrease in the von Frey paw withdrawal threshold in HFD compared to CD mice (CD = 1.35 ± 0.24 g vs. HFD = 0.68 ± 0.12 g; *p* < 0.001) that remained the same up to 16 weeks, consistent with the development of tactile allodynia. In contrast, the HFD + NR-fed mice had preserved NCVs, and the velocities were comparable to CD-fed mice (Figure 2). These findings are consistent with protection against peripheral neuropathy by dietary administration of NR. HFD + NR mice had normal tactile allodynia at 8 weeks compared to CD or CD + NR. Eight weeks after feeding mice with either a CD or HFD, mice were euthanized, and paw skins were examined for IENFD (Figure 2F). Skin biopsies showed a significant decrease in the IENFD of HFD mice (14.5 ± 2.5 fibers/mm) compared to CD mice (29.6 ± 4.4 fibers/mm; *p* < 0.001). On the other hand, in NR-treated mice fed with CD or HFD, the IENFD was the same as that in CD mice (HFD + NR 150 = 27.6 ± 3.5 fibers/mm: HFD + NR 300 = 30.8 ± 3.2 fibers/mm). These results suggest that dietary administration of NR protected against HFD-induced peripheral neuropathy.

### 2.6. Administration of NMN or NR Corrects STZ- or HFD-Induced Alterations in the NAD^+^ Metabolome

The targets of NR supplementation are presumed to affect whole body metabolism. Since sensory nerves die back in DPN, we focused on the DRG. We hypothesized that T1D and T2D might alter the NAD^+^ metabolome in DRG and contribute to neuropathy. We therefore employed LC-MS/MS to measure the NAD^+^ metabolome in DRG from freshly euthanized mice. We examined how NR administration influenced NAD^+^ biosynthesis in the brains and DRG of HFD mice by measuring the levels of NAD^+^ and its metabolites. After 2 months of feeding HFD mice with NR (300 mg/kg/day), the levels of NAD^+^ were increased (HFD = 1390 ± 81 vs. HFD + NR = 2140 ± 79 pmol/mg protein; *p* < 0.01). Smaller but statistically insignificant increases in the precursors (NR, NMN) and degradation product nicotinamide (NAM) were observed in NR-treated and HFD-fed mice (Table 4).

### 2.7. Dietary Supplementation with NR Maintains Mt Bioenergetic Function

**Figure 3:**
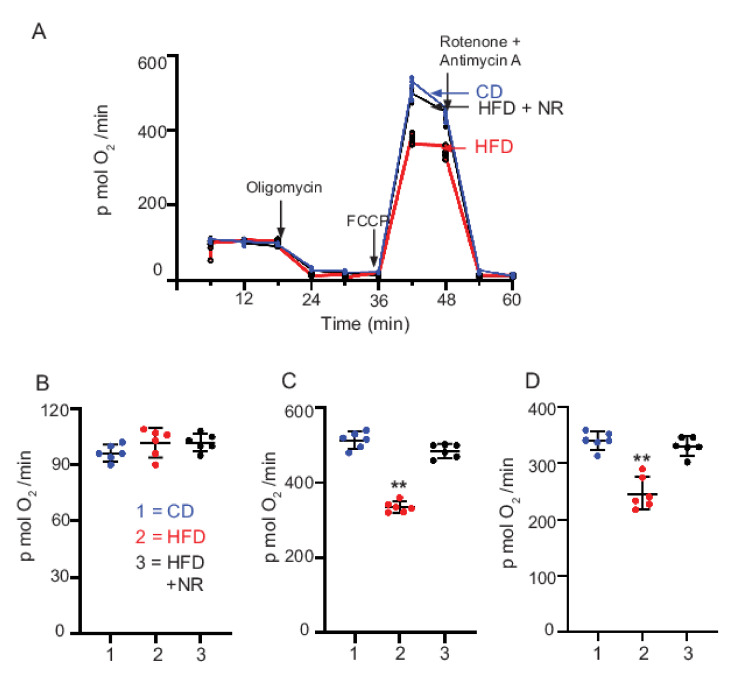
Mt respiration (*n* = 6/group). Measurement of Mt function in cultured DRG neurons using the XF24 analyzer. The oxygen consumption rate was measured at the basal level and with the subsequent and sequential addition of oligomycin, FCCP, and rotenone + antimycin A (AA) to DRG neurons cultured from CD, HFD, and HFD + NR 300 mg/kg mice (**A**). Levels of oxygen consumption rate were normalized per 6000 cells. From the oxygen consumption rate, basal respiration (**B**), maximal capacity respiration (**C**) and maximal reserve capacity (**D**) were calculated. The significance was calculated by ANOVA multiple comparison post hoc Tukey analysis. The statistical significance is indicated in the figure.; DRG neurons from HFD-fed mice compared to DRG neurons from CD and HFD + NR 300 mg/kg fed mice. ** *p* < 0.01.

To determine whether an NR-induced increase in NAD^+^ levels in DRG was able to regulate Mt bioenergetic function, we assessed the cellular bioenergetics profile of sensory neurons derived from DRG of age-matched CD, HFD, and HFD + NR mice. The oxygen consumption rate was measured in DRG neuronal culture prepared from 5-month-old adult mice using the Seahorse Biosciences XF24 analyzer (Figure 3). Basal, oligomycin-sensitive, and uncoupled respiration were measured by sequential addition with pyruvate and Mt complex inhibitors including, in order of injection, pyruvate, oligomycin (1.5 mM) to inhibit ATP synthase, FCCP (0.75 mM) to enable maximal rates of oxygen consumption, and a combination of rotenone and antimycin A to block respiratory electron flux at complexes I and III. The maximal oxygen consumption rate induced by the uncoupler FCCP in cultured neurons from CD mice (513 ± 22 pmol O_2_/min) was significantly higher (*p* < 0.01) compared to DRG neurons from HFD mice (335 ± 15 pmol O_2_/min), indicating impairment of maximal electron transport activity in the HFD-fed DRG. DRGs prepared from HFD + NR mice had a higher maximal oxygen consumption rate (485 ± 18 pmol O_2_/min). There was no difference in basal respiration between the groups. The spare respiratory capacity (maximal respiration minus basal respiration) in DRG neurons was decreased in HFD mice 247 ± 29 pmol O_2_/min compared to CD mice 340 ± 16 pmol O_2_/min, *p* < 0.001 and dietary administration of NR to HFD mice increased the spare reserve capacity to CD mice levels (330 ± 16 pmol O_2_/min). We interpret these results to suggest that although HFD caused a significant decrease in the maximal oxygen consumption rate, the Mt in DRG neurons from HFD + NR mice are better coupled and have a higher spare respiratory capacity.

## 3. Discussion

### 3.1. Diabetes and HFD Alter NAD^+^ Metabolic Pathways

The findings in this study show that improved Mt respiratory capacity caused by the administration of NMN or NR protected DRG neurons from STZ- and HFD-induced peripheral neuropathy. The administration of NR increased NAD^+^ levels in DRG.

The maintenance of NAD^+^ levels is central to axonal degeneration and regeneration pathways and to Mt homeostasis, including Mt oxidative phosphorylation, Mt biogenesis, and nuclear–mitochondrial communication [34,35]. The available evidence suggests that decreased synthesis and increased degradation of NAD^+^ in DRG are potential causes for axonal degeneration in DPN [19,33,36,37,38]. NAD^+^ can be synthesized in three ways: (1) de novo from tryptophan, (2) salvaged from NAM, or (3) from NMN or NR supplementation. In mammals, the salvage pathway is the predominant NAD^+^ biosynthetic pathway [39]. The pathway begins with the NAD^+^ degradation product, NAM, which combines with 5-phosphoribosyl pyrophosphate (PRPP) to form NMN catalyzed by the critical regulatory enzyme nicotinamide phosphoribosyl transferase (NAMPT) [39]. NMN is then adenylated by enzymes to NMN-adenyl transferase 1–3 to synthesize NAD ^+^. Therefore, the inhibition of NAMPT or NMNAT leads to reduced recycling of NAM and decreased NAD^+^ [40]. On the other hand, NR can be converted to NMN by the enzymes NRK1 and NRK2 [18,23].

### 3.2. Preservation of NAD^+^ Rescues Axonal Degeneration

In cultured DRG, the concentration of NAD^+^ was reported to decline after axotomy [15,21,36]. NAD^+^ is degraded by enzymes such as SIRT1, PARP1, and CD38 [18,23,27]. NAD^+^ is hydrolyzed by the enzyme SARM1 [41]. The activation of the enzyme DBC1 inhibits SIRT1 activity [27]. The activation of PARP1 or CD 38 or SARM1 or DBC1 promote axonal degeneration [22,23,24]. In contrast, the knockout of PARP1, CD38, SARM1, and DBC1 protect mice against HFD-induced neuropathy or chemotherapy-induced neuropathy [24,25,26,27,42,43]. Proteins that resynthesize NAD^+^ via the salvage pathway, such as NMNAT1–3, protect against axonal degeneration [28,29,30,31,32,42,44]. Among the NAD^+^ consuming enzymes, SIRT1 is unique because its activation protects against DPN whereas the inhibition of other NAD^+^ consuming proteins protect against DPN. We suggest that the protective pathway might involve inhibiting enzymes that would further degrade NAD^+^ by other NAD^+^-degrading enzymes but at the same time use the limited NAD^+^ to activate oxidative metabolism via SIRT1.

### 3.3. NAD^+^ Precursors Protect Alterations in Metabolic Syndrome

NR and NMN are better suited to increase NAD^+^ levels compared to other precursors of NAD^+^ such as nicotinamide (NAM) and nicotinic acid/niacin (NA) [45,46]. Importantly, clinical trials using NR and NMN show that oral administration of NR or NMN was safe and effectively metabolized in healthy men without causing any significant deleterious effects [47,48]. NAM has been shown to reverse nerve conduction and neurovascular effects in experimental diabetes and reduce oxidative stress after two weeks of treatment [49]. Prolonged exposure to NAM may lead to impaired β-cell function, a reduction in cell growth [50,51], and the inhibition of sirtuins [52,53]. Additionally, methylation of NAM by the enzyme NAM-N-methyl transferase causes the accumulation of 1-methyl-nicotinamide (MeNAM), which in turn causes the depletion of methyl donors and the accumulation of fat in adipose tissue [54,55,56]. Knockout of NMNT gene, knockdown of NMNT expression with siRNA, and chemical inhibition of NMNT activity promote the NAD^+^ salvage pathway via the enzyme NAMPT and by increasing NMN to resist HFD-induced obesity [54,55,56]. NAD^+^ cannot be administered directly due to its toxic effects that include serious hyperglycemia lasting for hours. Another source of NAD^+^ is NA. The metabolic factors resulting in DPN are complex but include both hyperglycemia and hyperlipidemia [1,2,57]. Nicotinic Acid (NA) is used to treat dyslipidemia by lowering the level of triglycerides and LDL and raising the level of HDL [58]. Inducing diabetes with STZ or feeding with an HFD caused a significant increase in plasma glucose, cholesterol, triglycerides, and NEFA. The blood results showed similar increases in glucose levels between HFD and HFD + NR in mice or between STZ and STZ + NMN in mice and rats, suggesting that NR and NMN do not reduce the glucotoxicity induced by STZ or an HFD. Furthermore, NR treatment does not affect insulin resistance (Table 3).

The major changes observed in HFD + NR compared to HFD or STZ + NMN compared to STZ were that both NMN and NR prevented increases in triglycerides and NEFA levels (Table 1, Table 2 and Table 3), suggesting that both NMN and NR may promote lipid oxidation [59,60,61]. NA often leads to severe flushing, mediated by the binding of NA to the GPR109A receptor [62,63]. Therefore, NAD^+^ precursors that do not activate GPR109A [64] but still increase NAD^+^ levels, such as NR and NMN, would be clinically useful. Our results on NAD^+^ metabolites showed that 2 months of NR administration increases NAM to a lesser extent in HFD-fed mice (200 ± 23 p mol/mg protein; Table 3) than in CD-fed mice (270 ± 14 p mol/mg protein). A HFD is associated with a decrease in NAD^+^ levels, potentially suggesting that more NR is directed toward the synthesis of NAD^+^ than toward the degradation product NAM. Furthermore, both NMN and NR are better suited to increase NAD^+^ levels because the conversion of NMN to NAD^+^ requires one ATP and the conversion of NR to NAD^+^ requires two ATPs. In contrast, the conversion of NAM to NAD^+^ requires four ATPs and NA to NAD^+^ requires five ATPs [65].

### 3.4. HFD-Induced Decrease in Mt Respiration Capacity Was Rescued by NR Administration

Several research groups have shown that the effect of NAD^+^ precursors is mainly through the enhancement of Sirtuin pathways. The administration of NR elevates NAD^+^ levels and increases the activity of nuclear and Mt NAD^+^-dependent protein lysine deacetylases, including sirtuins, SIRT1, and SIRT3. Likewise, NMN treatment ameliorates HFD-induced insulin resistance by restoring NAD^+^ biosynthesis and SIRT1/SIRT3 activity [40,45]. SIRT1 promotes the deacetylation of PGC-1α that in turn promotes Mt biogenesis. In support of this, the administration of NR has been shown to enrich the Mt content in several tissues.

The estimated total intracellular content of NAD^+^ is in the 0.2 to 0.5 mM range [66,67], which lies within the estimated *K*m values of SIRT1 for NAD^+^. We have shown that NAD^+^ in the peripheral DRG neurons of diabetic rats and HFD-fed mice is decreased and levels of NAD^+^ lie in the lower end of the Km value of the SIRT1 enzyme. This links depleted NAD^+^ to DPN and impaired SIRT1 activation. Our results show that the NR-induced increase in NAD^+^ levels in DRG neurons were able to stimulate Mt’s bioenergetic function. Our studies suggest that NR/NMN administration enhances the SIRT1/PGC-1α axis in neurons and protects against axonal degeneration in diabetic and HFD-fed rodents. The bioenergetics profile of DRG from HFD-fed mice show a decrease in maximal and spare reserve capacity, rendering Mt more vulnerable to stress. This decrease in spare reserve capacity was normalized in DRG neurons from HFD + NR mice. Our recent results also showed that the bioenergetics profile of hippocampal Mt from NMN administration show a higher maximal and spare reserve capacity in both non-diabetic and diabetic rats. This higher reserve capacity is consistent with the idea that increasing NAD^+^ levels and/or increasing SIRT1 protein/activity levels primes neuronal Mt to overcome the failure of bioenergetic function induced by an increased glucose load [68,69,70]. SIRT1 is central to the regulation of both Mt function and neuronal preservation [71,72]. For example, SIRT1 uses NAD^+^ to deacetylate proteins and the administration of NMN prevents the depletion of cellular NAD^+^ to maintain cellular homeostasis. This occurs by regulating the deacetylation of crucial proteins to maintain quality control via Mt metabolism.

The questions not addressed in this manuscript are (1) can the administration of NMN or NR reverse DPN? Our results do show that the administration of NMN or NR reverse DPN impairments. The results of those experiments will be addressed in a separate manuscript; (2) what is the optimal dose of NR or NMN for DPN treatment? We tried two different doses of NMN IP, 50 and 100 mg/kg, and two different doses of NR in the diet, 150 and 300 mg/kg. We did not find statistically significant differences in test measurements between these two doses (Group 4 Vs Group 5 in Table 1 to Table 3 was not statistically different). Interestingly, NAD^+^ level showed an increase with NR 300 mg/kg treatment compared to 150 mg/kg treatment. However, we did not see significant effects on measures of DPN, suggesting that an increase in NAD^+^ to basal level (set point) is sufficient to protect against DPN. Further experiments with varying doses are needed to determine the appropriate therapeutic dose; (3) The study did not directly address the effect of NMN or NR on the stress–habituation response. Both experimental (NMN in saline) and control animals (saline) received the same number of injections at the same volume at the same time of day using an identical technique. It is therefore likely that the stress–habituation response would be similar between the experimental and control animals; however, this was not directly tested. Similarly, the diet consumed by experimental and control animals was identical except for the use of NR in the experimental diet.

In summary, our results show that IP administration of NMN or dietary administration of NR prevented the potential pathogenic pathway in diabetic neuropathy. The study suggests that medications that prevent NAD^+^ depletion induced by diabetes mellitus and molecules that activate SIRT may provide a therapeutic intervention for diabetic neurodegeneration.

## 4. Materials and Methods

### 4.1. Diabetes Induction with STZ

All animal protocols followed the National Institutes of Health (NIH) Guide for the Care and Use of Laboratory Animals and were approved by the Institutional Animal Care and Use Committee (IACUC Protocol # 0421009, approved date: 17 May 2021). Sprague Dawley (SD) rats were purchased from Charles River, Wilmington, MA, USA. The animals were adapted to the laboratory environment for 1 week before the experiments. Each animal at 12 weeks old (weighing 264 ± 18 g) was injected by intraperitoneal injection (IP) with a single dose of STZ (62 mg/kg; Sigma, St. Louis, MO, USA) dissolved in 0.01 mol/L citric acid solution (pH = 4.5). Control rats received the solvent injected IP. The animals were then housed individually in a temperature and humidity-controlled environment on a 12 h light/dark cycle. To prevent hypoglycemia, the STZ-treated rats had free access to water with 5% dextrose during the first 24 h after STZ injection. The fasting blood glucose levels in the STZ-treated and control animals were measured 3 days after the treatments. STZ treated animals with a fasting blood glucose level of 300 mg/dL or 11 mmol/L were used. Five-month-old C57BL6 mice were used for the STZ injections. Animals that had a weight loss of more than 20% were euthanized and not included in the study. Sixty percent of the animals fulfilled the criteria and were used. After confirmation of diabetes, NMN was administered. NMN was dissolved in saline and injected into diabetic animals at a dose of 50 mg/kg or 100 mg/kg on alternate days (Monday, Wednesday, and Friday) for 3 months. Control animals were administered with either saline or NMN at a dose of 100 mg/kg.

### 4.2. T2D Diabetes Induction with HFD

Wild-type C57BL6 mice (3 months old) were fed with a CD or HFD. The CD (Harlan-Teklad #2018) contained 6.2% fat (18% calories from fat), 18.6% protein (24% calories from protein), and 44.2% carbohydrate (58% calories from carbohydrate). The HFD (Bio-Serv #F3282) contained 36% fat (60% calories from fat), 20.5% protein (15% calories from protein), and 37.5% carbohydrate (26% calories from carbohydrate). One-week after HFD-diet feeding, NMN was administered. Nicotinamide mononucleotide (NMN) was dissolved in saline and injected IP into diabetic rats at a dose of 50 mg/kg or 100 mg/kg on alternate days (Monday, Wednesday, and Friday) for 3 months. The control animals were injected IP with saline on alternate days. Chronic IP administration of NMN at a dose of 500 mg/kg/day for 10 days has been previously found to be safe and effective [17]. The total length of the HFD study was 30 weeks.

### 4.3. Experimental Design

**Experiment Design in Rats (Figure 1 and Table 1):** Thirty male SD rats were randomly assigned to 5 groups of 6 per group. Five groups of rats were used; 1. non-diabetic; 2. diabetic; 3. non-diabetic + NMN (100 mg/kg); 4. diabetic + NMN (50 mg/kg); and 5. diabetic + NMN (100 mg/kg).

Group 1 rats, non-diabetic (non-Dia), were injected intraperitoneally (IP) with saline on alternate days for 2 months; Group 2 rats were injected IP with NMN in saline on alternate days for 2 months at a dose of 100 mg/kg; Group 3 rats were injected IP with a single dose of STZ at a dose of 65 mg/kg to induce T1D; Group 4 rats were made diabetic with STZ injection and injected IP with NMN on alternate days for 2 months at a dose of 50 mg/kg; and Group 5 rats were made diabetic with STZ injection and injected IP with NMN on alternate days for 2 months at a dose of 100 mg/kg.

**Experimental Design in Mice Given NMN (Table 2):** We tested whether NMN injection could prevent peripheral neuropathy in 5-month-old C57BL6 mice. There were 5 groups of mice in categories identical to the rat groups. The dose of NMN injected IP was the same as that of rats, i.e., 50 mg/kg and 100 mg/kg. Considering the surface area of rats and mice, the dose of 100 mg/kg in rats would correspond to 50 mg/kg in mice. The dose of 100 mg/kg in mice would represent a higher dose. T1D was induced by the administration of STZ. One week before euthanasia, mice were tested for SMNCV, TML, TSNCV, mechanical allodynia by von Frey filaments, and thermal sensitivity by the Hargreaves test. IP-GTT were performed on fasted mice as previously described [11]. Mice were euthanized, blood was collected, hind paws were fixed, and IENFD was quantified following PGP 9.5 protein staining.

**Experimental Design in Mice Given NR (Figure 2):** Three-month-old C57BL6 mice were used. NR was mixed with the HFD at 2 doses of 150 mg and 300 mg/kg and fed for 2 months. This would correspond to a human dose of 1–2 g/day. There were 5 groups of mice: CD (Group 1); CD + NR (300 mg/kg; Group 2); HFD (Group 3); HFD + NR (150 mg/kg; Group 4); and HFD + NR (300 mg/kg; Group 5).

### 4.4. Neuropathy Measurements

Peripheral neuropathy was tested following the guidelines of the European diabetic neuropathy study group [73]. Mechanical allodynia was assessed using Somedic von Frey monofilaments [74,75]. Briefly, ordinal numbers were applied gently on the fat part of both plantar heels until the filament started to bend and this was maintained for ~2 s. The threshold was defined as the minimal bending force of the thinnest filament sensed by the animal in an ascending and descending series. A withdrawal response was considered valid only if the hind paw was completely removed from the platform. The Hargreaves test was used to test thermal nociception, which assesses small nerve fiber function [11]. Light from a halogen bulb lamp was delivered to the plantar surface of the mouse hind paw through the base of the glass panel to induce the heat stimuli. The time taken for the mouse to lift or lick its hind paw was recorded automatically. Nerve conduction studies were performed as described in [11,75]. The animals were provided with thermal support and isoflurane anesthesia via a nose cone (1–2%). NCV studies were performed in the left hind limb and in the tail using platinum electrodes, placed adjacent to the nerve, using a 60–80 mA square wave stimulus for 0.1–0.3 ms to obtain near nerve recordings. The G1 (active) and G2 (indifferent) recording electrodes were separated by 10 mm. In the left hind limb, distal latency to the dorsum of the paw and proximal fibular/sciatic conduction velocity were obtained. Tail NCVs were recorded over a 4 cm distance measured from the base of the tail. Orthodromic motor conduction velocities were obtained by recording at the tip of the tail and stimulating with the cathode proximal to the G1 recording electrode. Orthodromic sensory tail conduction velocities were obtained by placing G1 at the base of the tail and stimulating 4 cm distally. Sensory responses were averaged until the sensory nerve action potential response was stable. Tail and limb near nerve temperatures were maintained at 32–33 °C. The onset latency and peak amplitude were measured. IENFD was measured using PGP9.5 antibody staining in a blinded fashion, as previously described [11,74]. To delineate fiber crossing at the dermo/epidermal junction, slides were counterstained by dipping in eosin (Sigma-Aldrich Eosin Y solution HT110316). IENFD was calculated (as fibers/mm) by the number of complete baseline crossings of nerve fibers at the dermo-epidermal junction divided by the measured length of the epidermal surface using standardized validated methods [76,77].

### 4.5. Mt Respiration

DRG neurons from adult mice were cultured as described in [11,78]. In brief, the well of the Seahorse plate was coated with 250 µL poly-L-lysine (PLL; 100 mg/mL in water) and laminin (200 µL of 2.5 mg/mL in PBS). DRGs were collected from CD, HFD, and HFD + NR mice and cultured. After 24 h of incubation, basal, oligomycin-sensitive, and uncoupled respiration were measured by sequential addition with pyruvate, oligomycin (1.5 mM) to inhibit ATP synthase, FCCP (0.75 mM) to enable maximal rates of oxygen consumption, and a combination of rotenone (1 mM) and antimycin A (1 mM) to block Mt respiration. The basal level of oxygen consumption, oligomycin-sensitive respiration, maximal respiration capacity, and non-Mt oxygen consumption were measured [11].

### 4.6. Quantification of NAD^+^

A solution of 0.5 N perchloric acid four times the weight of the DRG tissue was added to the frozen tissue. These samples were homogenized using a Qiagen tissue lyzer. A 10 μL aliquot of the internal standard solution (50 µg/mL in water) was added to the tissue homogenate. The sample was centrifuged at 1600× *g* for 10 min at room temperature. Next, 50 μL of supernatant was transferred to a clean 96-deep-well plate and diluted further with 150 μL of 5 mM ammonium formate and the NAD^+^ level was quantified following the protocol of Liang et al. [79]. Briefly, NAD^+^ analysis in tissue samples was carried out with QTRAP^®^ 5500 mass spectrometer (AB Sciex, Framingham, MA, USA) equipped with a turbo-electrospray interface in positive ionization mode. The aqueous mobile phase was water with 0.1% formic acid and the organic mobile phase was acetonitrile with 0.1% formic acid. The gradient was 0% formic acid for the first 0.1 min, and then increased to 30% formic acid in 0.9 min, decreased to 0% formic acid within 0.1 min, and maintained at 0% formic acid for another 0.4 min. The flow rate was 0.8 mL/min and the cycle time (injection to injection including instrument delays) was approximately 1.8 min. A volume of 1–3 μL of the final extract was injected onto the analytical dC18 column (100 × 2.1 mm, 3 μm, Waters, Milford, MA, USA).

### 4.7. Statistical Analysis

A comparison of dependent variables was performed on transformed data using factorial ANOVA with a post hoc Tukey test to determine significant differences between groups. Individual comparisons were made using Student’s *t*-test, assuming unequal variances as previously described [80]. The associations between the Mt function and measures of neuropathy (NCV and mechanical allodynia) were evaluated using Spearman correlation statistics.

## Figures and Tables

**Table 1:** Metabolic End Points After 2 Months of Treatment in Non-Diabetic (Non-Dia), NMN Treated Non-Diabetic (Non-Dia + NMN), Diabetic (STZ), NMN Treated Diabetic (STZ + NMN) Rats. NS = Not Significant.

Parameters	WT					Significance			
	Non-Dia (*n* = 6)	Non-Dia + NMN (100 mg/kg) (*n* = 6)	STZ (*n* = 6)	STZ + NMN (50 mg/kg) (*n* = 6)	STZ + NMN (100 mg /kg) *(n* = 6)	1 vs. 2	1 vs. 3	3 vs. 4	3 vs. 5
Group #	1	2	3	4	5				
Body Weight (g)	450 ± 17	431 ± 19	346 ± 14	345 ± 13	331 ± 14	NS	<0.001	NS	NS
Plasma Glucose (mg/dL)	132 ± 18	128 ± 17	405 ± 22	399 ± 13	388 ± 15	NS	<0.001	NS	NS
HbA1c%	5.4 ± 1	5.4 ± 1	16.7 ± 4.1	15.8 ± 2.6	15.3 ± 2.9	NS	<0.01	NS	NS
Insulin (mµ/mL)	8.3 ± 2	8 ± 2	1.2 ± 0.7	1.3 ± 0.4	1.6 ± 0.3	NS	<0.001	NS	NS
Total Cholesterol (mg/dL)	68 ± 13	59 ± 11	173 ± 14	133 ± 71	139 ± 48	NS	<0.01	NS	NS
Triglycerides (mg/dL)	94 ± 11	96 ± 9	493 ± 23	376 ± 22	361 ± 19	NS	<0.001	<0.05	<0.05
HDL (mg/dL)	79 ± 4	78 ± 5	128 ± 21	119 ± 15	115 ± 16	NS	<0.001	NS	NS
LDL (mg/dL)	13 ± 5	10 ± 4	63 ± 5	64 ± 9	60 ± 9	NS	<0.01	NS	NS
NEFA (mM)	2.5 ± 0.8	3.1 ± 0.4	6.6 ± 1.8	3.6 ± 1	3.8 ± 1	NS	<0.001	<0.05	<0.05

**Table 2:** Metabolic End Points After 2 Months of Treatment in Non-Diabetic (Non-Dia), Non-Dia + NMN (100 mg/kg intraperitoneal injection on alternate days), Diabetic (STZ), STZ + NMN (50 mg/kg intraperitoneal injection on alternate days) and STZ + NMN (100 mg/kg intraperitoneal injection on alternate days) in C57BL6 Mice. NS = Not Significant.

Parameters	WT					Significance			
	Non-Dia (*n* = 6)	Non-Dia + NMN (100 mg/kg) (*n* =6)	STZ (*n* = 6)	STZ + NMN (50 mg/kg) (*n* = 6)	STZ + NMN (100 mg /kg) (*n* = 6)	1 vs. 2	1 vs. 3	3 vs. 4	3 vs. 5
Group #	1	2	3	4	5				
Body Weight (g)	30 ± 2	31 ± 3	26 ± 4	25 ± 3	21 ± 4	NS	<0.01	NS	NS
Plasma Glucose (mg/dL)	105 ± 12	108 ± 11	405 ± 32	385 ± 33	388 ± 35	NS	<0.001	NS	NS
HbA1c%	5.4 ± 1	5.4 ± 1	15.7 ± 3.1	15.8 ± 3.6	15.3 ± 3.4	NS	<0.001	NS	NS
Insulin (µg/L)	0.8 ± 0.2	0.8 ± 0.2	0.2 ± 0.05	0.18 ± 0.04	0.16 ± 0.03	NS	<0.001	NS	NS
Total Cholesterol (mg/dL)	78 ± 13	75 ± 21	173 ± 42	163 ± 21	169 ± 28	NS	<0.01	NS	NS
Triglycerides (mg/dL)	44 ± 8	36 ± 9	93 ± 13	66 ± 10	61 ± 10	NS	<0.001	<0.05	<0.05
HDL (mg/dL)	79 ± 4	78 ± 5	108 ± 21	99 ± 15	95 ± 16	NS	<0.01	NS	NS
LDL (mg/dL)	43 ± 5	50 ± 4	83 ± 5	84 ± 9	90 ± 9	NS	<0.01	NS	NS
NEFA (mM)	2.5 ± 0.8	3.1 ± 0.4	6.4 ± 1.4	3.6 ± 1	3.6 ± 0.8	NS	<0.001	<0.05	<0.05
Sciatic MNCV (m/s)	55 ± 7	53 ± 6	26 ± 6	42.2 ± 7	46 ± 6.5	NS	<0.001	<0.001	<0.001
Tail MNCV (m/s)	45 ± 4	43 ± 6	24 ± 3	46 ± 4.5	48 ± 5.3	NS	<0.001	<0.001	<0.001
Tail SNCV (m/s)	41 ± 3	43 ± 3	27 ± 5	46 ± 7	48 ± 6	NS	<0.001	<0.001	<0.001
Von Frey Mechanical Allodynia (g)	1.2 ± 0.2	1.3 ± 0.3	0.6 ± 0.1	1.0 ± 0.2	1.2 ± 0.3	NS	<0.001	<0.01	<0.001
Hargreaves Thermal Response (sec)	12 ± 2	14 ± 3	22 ± 4	14 ± 3	12 ± 3	NS	<0.001	<0.001	<0.001
IENFD (# / mm)	25 ± 3	23 ± 4	12 ± 2	23 ± 3	24 ± 3	NS	<0.001	<0.001	<0.001

**Table 3:** Metabolic End Points After 2 Months in WT mice Fed With CD, CD + NR (300 mg/kg), HFD, HFD + NR (150 and 300 mg/kg). NS = Not Significant.

Parameters	WT					Significance			
	CD (*n* = 8)	CD + NR (300 mg/kg) (*n* = 8)	HFD (*n* = 8)	HFD + NR (150 mg/kg) (*n* = 8)	HFD + NR (300 mg/kg) (*n* = 8)	1 vs. 2	1 vs. 3	3 vs. 4	3 vs. 5
Group #	1	2	3	4	5				
Body Weight (g)	30 ± 2	31 ± 3	46 ± 4	45 ± 3	41 ± 4	NS	<0.001	NS	NS
Plasma Glucose (mg/dL)	135 ± 18	148 ± 17	205 ± 22	185 ± 13	188 ± 15	NS	<0.001	NS	NS
HbA1c%	5.4 ± 1	5.4 ± 1	6.7 ± 1.1	5.3 ± 0.6	5.8 ± 0.9	NS	<0.001	<0.001	<0.01
Insulin (µg/L)	0.8 ± 0.2	0.8 ± 0.2	4 ± 0.5	3.8± 0.4	3.6 ± 0.3	NS	<0.001	NS	NS
Total Cholesterol (mg/dL)	152 ± 32	115 ± 12	273 ± 42	233 ± 51	249 ± 21	NS	<0.001	<0.05	<0.05
Triglycerides (mg/dL)	144 ± 18	136 ± 16	241 ± 32	142 ± 28	96 ± 16	NS	<0.001	<0.01	<0.001
HDL Cholesterol (mg/dL)	142 ± 14	130 ± 14	256 ± 29	169 ± 15	203 ± 26	NS	<0.001	<0.001	<0.05
Non-HDL Cholesterol (mg/dL)	10 ± 3	12 ± 3	15 ± 6	14 ± 2	10 ± 3	NS	NS	NS	NS
NEFA (mM)	3 ± 0.29	3.1 ± 0.4	5.9 ± 1	3.6 ± 0.7	4.8 ± 1	NS	<0.001	<0.001	<0.05
GTT-AUC (mg*min/dL X 10^3^)	3 ± 0.29	3.6 ± 0.46	6.1 ± 1	6.2 ± 1	5.9 ± 1.2	NS	<0.001	NS	NS

**Table 4:** NR, NMN, and NAD^+^ Metabolites From DRG Extracts in CD, HFD or After 2 Months of NR Treatment. NS = Not Significant.

Parameters. (p mol/mg Protein)	WT					Significance			
	CD (*n* = 11)	CD + NR (300 mg/kg) (*n* = 11)	HFD (*n* = 11)	HFD + NR (150 mg/kg) (*n* = 11)	HFD + NR (300 mg/kg) *(n* = 11)	1 vs. 2	1 vs. 3	3 vs. 4	3 vs. 5
Group #	1	2	3	4	5				
NAD^+^	1830 ± 94	2010 ± 98	1390 ± 81	1880 ± 72	2140 ± 79	NS	<0.001	<0.05	<0.01
NMN	3.8 ± 0.6	4.4 ± 0.7	4.1 ± 0.8	4.6 ± 0.9	4.7 ± 0.9	NS	NS	NS	NS
NR	3.8 ± 0.7	5.9 ± 0.8	3.6 ± 0.8	5.65 ± 0.5	5.6 ± 0.6	<0.05	NS	<0.05	<0.05
Nam	130 ± 13	270 ± 14	190 ± 24	200 ± 23	210 ± 26	<0.005	<0.05	NS	NS

## Data Availability

Data is available up on request.

## Supplementary Materials

Supplementary materials can be found at https://www.mdpi.com/article/10.3390/ijms23094887/s1.

## References

[1] Feldman E.L., Callaghan B.C., Pop-Busui R., Zochodne D.W., Wright D.E., Bennett D.L., Bril V., Russell J.W., Viswanathan V. (2019). Diabetic neuropathy. Nat. Rev. Dis. Primers.

[2] Zilliox L.A., Russell J.W. (2019). Physical activity and dietary interventions in diabetic neuropathy: A systematic review. Clin. Auton. Res..

[3] Rumora A.E., Lentz S.I., Hinder L.M., Jackson S.W., Valesano A., Levinson G.E., Feldman E.L. (2018). Dyslipidemia impairs mitochondrial trafficking and function in sensory neurons. FASEB J..

[4] Pop-Busui R., Boulton A.J., Feldman E.L., Bril V., Freeman R., Malik R.A., Sosenko J.M., Ziegler D. (2017). Diabetic Neuropathy: A Position Statement by the American Diabetes Association. Diabetes Care.

[5] Eid S., Sas K.M., Abcouwer S.F., Feldman E.L., Gardner T.W., Pennathur S., Fort P.E. (2019). New insights into the mechanisms of diabetic complications: Role of lipids and lipid metabolism. Diabetologia.

[6] Viader A., Golden J.P., Baloh R.H., Schmidt R.E., Hunter D.A., Milbrandt J. (2011). Schwann cell mitochondrial metabolism supports long-term axonal survival and peripheral nerve function. J. Neurosci..

[7] Viader A., Sasaki Y., Kim S., Strickland A., Workman C.S., Yang K., Gross R.W., Milbrandt J. (2013). Aberrant Schwann cell lipid metabolism linked to mitochondrial deficits leads to axon degeneration and neuropathy. Neuron.

[8] Choi J., Ravipati A., Nimmagadda V., Schubert M., Castellani R.J., Russell J.W. (2014). Potential roles of PINK1 for increased PGC-1alpha-mediated mitochondrial fatty acid oxidation and their associations with Alzheimer disease and diabetes. Mitochondrion.

[9] Feldman E.L., Nave K.A., Jensen T.S., Bennett D.L.H. (2017). New Horizons in Diabetic Neuropathy: Mechanisms, Bioenergetics, and Pain. Neuron.

[10] Rumora A.E., Savelieff M.G., Sakowski S.A., Feldman E.L. (2019). Disorders of mitochondrial dynamics in peripheral neuropathy: Clues from hereditary neuropathy and diabetes. Int. Rev. Neurobiol..

[11] Chandrasekaran K., Salimian M., Konduru S.R., Choi J., Kumar P., Long A., Klimova N., Ho C.Y., Kristian T., Russell J.W. (2019). Overexpression of Sirtuin 1 protein in neurons prevents and reverses experimental diabetic neuropathy. Brain.

[12] Chandrasekaran K., Anjaneyulu M., Choi J., Kumar P., Salimian M., Ho C.Y., Russell J.W. (2019). Role of mitochondria in diabetic peripheral neuropathy: Influencing the NAD(+)-dependent SIRT1-PGC-1alpha-TFAM pathway. Int. Rev. Neurobiol..

[13] Demare S., Kothari A., Calcutt N.A., Fernyhough P. (2021). Metformin as a potential therapeutic for neurological disease: Mobilizing AMPK to repair the nervous system. Expert Rev. Neurother..

[14] Schartner E., Sabbir M.G., Saleh A., Silva R.V., Roy Chowdhury S., Smith D.R., Fernyhough P. (2018). High glucose concentration suppresses a SIRT2 regulated pathway that enhances neurite outgrowth in cultured adult sensory neurons. Exp. Neurol..

[15] Sasaki Y. (2019). Metabolic aspects of neuronal degeneration: From a NAD(+) point of view. Neurosci. Res..

[16] Chandrasekaran K., Choi J., Arvas M.I., Salimian M., Singh S., Xu S., Gullapalli R.P., Kristian T., Russell J.W. (2020). Nicotinamide Mononucleotide Administration Prevents Experimental Diabetes-Induced Cognitive Impairment and Loss of Hippocampal Neurons. Int. J. Mol. Sci..

[17] Fang E.F., Lautrup S., Hou Y., Demarest T.G., Croteau D.L., Mattson M.P., Bohr V.A. (2017). NAD(+) in Aging: Molecular Mechanisms and Translational Implications. Trends Mol. Med..

[18] Yoshino J., Baur J.A., Imai S.I. (2018). NAD(+) Intermediates: The Biology and Therapeutic Potential of NMN and NR. Cell Metab..

[19] Trammell S.A., Weidemann B.J., Chadda A., Yorek M.S., Holmes A., Coppey L.J., Obrosov A., Kardon R.H., Yorek M.A., Brenner C. (2016). Nicotinamide Riboside Opposes Type 2 Diabetes and Neuropathy in Mice. Sci. Rep..

[20] Gerdts J., Summers D.W., Milbrandt J., DiAntonio A. (2016). Axon Self-Destruction: New Links among SARM1, MAPKs, and NAD+ Metabolism. Neuron.

[21] Coleman M.P., Hoke A. (2020). Programmed axon degeneration: From mouse to mechanism to medicine. Nat. Rev. Neurosci..

[22] Osterloh J.M., Yang J., Rooney T.M., Fox A.N., Adalbert R., Powell E.H., Sheehan A.E., Avery M.A., Hackett R., Logan M.A. (2012). dSarm/Sarm1 is required for activation of an injury-induced axon death pathway. Science.

[23] Geisler S., Doan R.A., Strickland A., Huang X., Milbrandt J., DiAntonio A. (2016). Prevention of vincristine-induced peripheral neuropathy by genetic deletion of SARM1 in mice. Brain.

[24] Turkiew E., Falconer D., Reed N., Hoke A. (2017). Deletion of Sarm1 gene is neuroprotective in two models of peripheral neuropathy. J. Peripher. Nerv. Syst..

[25] Barbosa M.T., Soares S.M., Novak C.M., Sinclair D., Levine J.A., Aksoy P., Chini E.N. (2007). The enzyme CD38 (a NAD glycohydrolase, EC 3.2.2.5) is necessary for the development of diet-induced obesity. FASEB J..

[26] Obrosova I.G., Xu W., Lyzogubov V.V., Ilnytska O., Mashtalir N., Vareniuk I., Pavlov I.A., Zhang J., Slusher B., Drel V.R. (2008). PARP inhibition or gene deficiency counteracts intraepidermal nerve fiber loss and neuropathic pain in advanced diabetic neuropathy. Free Radic. Biol. Med..

[27] Escande C., Chini C.C., Nin V., Dykhouse K.M., Novak C.M., Levine J., van Deursen J., Gores G.J., Chen J., Lou Z. (2010). Deleted in breast cancer-1 regulates SIRT1 activity and contributes to high-fat diet-induced liver steatosis in mice. J. Clin. Investig..

[28] Sasaki Y., Araki T., Milbrandt J. (2006). Stimulation of nicotinamide adenine dinucleotide biosynthetic pathways delays axonal degeneration after axotomy. J. Neurosci..

[29] Press C., Milbrandt J. (2008). Nmnat delays axonal degeneration caused by mitochondrial and oxidative stress. J. Neurosci..

[30] Yahata N., Yuasa S., Araki T. (2009). Nicotinamide mononucleotide adenylyltransferase expression in mitochondrial matrix delays Wallerian degeneration. J. Neurosci..

[31] Babetto E., Beirowski B., Janeckova L., Brown R., Gilley J., Thomson D., Ribchester R.R., Coleman M.P. (2010). Targeting NMNAT1 to axons and synapses transforms its neuroprotective potency in vivo. J. Neurosci..

[32] Di Stefano M., Loreto A., Orsomando G., Mori V., Zamporlini F., Hulse R.P., Webster J., Donaldson L.F., Gering M., Raffaelli N. (2017). NMN Deamidase Delays Wallerian Degeneration and Rescues Axonal Defects Caused by NMNAT2 Deficiency In Vivo. Curr. Biol..

[33] Ilnytska O., Lyzogubov V.V., Stevens M.J., Drel V.R., Mashtalir N., Pacher P., Yorek M.A., Obrosova I.G. (2006). Poly(ADP-ribose) polymerase inhibition alleviates experimental diabetic sensory neuropathy. Diabetes.

[34] Gomes A.P., Price N.L., Ling A.J., Moslehi J.J., Montgomery M.K., Rajman L., White J.P., Teodoro J.S., Wrann C.D., Hubbard B.P. (2013). Declining NAD(+) induces a pseudohypoxic state disrupting nuclear-mitochondrial communication during aging. Cell.

[35] Mouchiroud L., Houtkooper R.H., Auwerx J. (2013). NAD(+) metabolism: A therapeutic target for age-related metabolic disease. Crit. Rev. Biochem. Mol. Biol..

[36] Li F., Drel V.R., Szabo C., Stevens M.J., Obrosova I.G. (2005). Low-dose poly(ADP-ribose) polymerase inhibitor-containing combination therapies reverse early peripheral diabetic neuropathy. Diabetes.

[37] Li F., Szabo C., Pacher P., Southan G.J., Abatan O.I., Charniauskaya T., Stevens M.J., Obrosova I.G. (2004). Evaluation of orally active poly(ADP-ribose) polymerase inhibitor in streptozotocin-diabetic rat model of early peripheral neuropathy. Diabetologia.

[38] Cheng Y., Liu J., Luan Y., Liu Z., Lai H., Zhong W., Yang Y., Yu H., Feng N., Wang H. (2019). Sarm1 Gene Deficiency Attenuates Diabetic Peripheral Neuropathy in Mice. Diabetes.

[39] Imai S., Yoshino J. (2013). The importance of NAMPT/NAD/SIRT1 in the systemic regulation of metabolism and ageing. Diabetes Obes. Metab..

[40] Yoshino J., Mills K.F., Yoon M.J., Imai S. (2011). Nicotinamide mononucleotide, a key NAD(+) intermediate, treats the pathophysiology of diet- and age-induced diabetes in mice. Cell Metab..

[41] Gerdts J., Brace E.J., Sasaki Y., DiAntonio A., Milbrandt J. (2015). SARM1 activation triggers axon degeneration locally via NAD(+) destruction. Science.

[42] Gilley J., Coleman M.P. (2010). Endogenous Nmnat2 is an essential survival factor for maintenance of healthy axons. PLoS Biol..

[43] Gilley J., Ribchester R.R., Coleman M.P. (2017). Sarm1 Deletion, but Not Wld(S), Confers Lifelong Rescue in a Mouse Model of Severe Axonopathy. Cell Rep..

[44] Sasaki Y., Vohra B.P., Baloh R.H., Milbrandt J. (2009). Transgenic mice expressing the Nmnat1 protein manifest robust delay in axonal degeneration in vivo. J. Neurosci..

[45] Canto C., Houtkooper R.H., Pirinen E., Youn D.Y., Oosterveer M.H., Cen Y., Fernandez-Marcos P.J., Yamamoto H., Andreux P.A., Cettour-Rose P. (2012). The NAD(+) precursor nicotinamide riboside enhances oxidative metabolism and protects against high-fat diet-induced obesity. Cell Metab..

[46] Gille A., Bodor E.T., Ahmed K., Offermanns S. (2008). Nicotinic acid: Pharmacological effects and mechanisms of action. Annu. Rev. Pharmacol. Toxicol..

[47] Conze D., Brenner C., Kruger C.L. (2019). Safety and Metabolism of Long-term Administration of NIAGEN (Nicotinamide Riboside Chloride) in a Randomized, Double-Blind, Placebo-controlled Clinical Trial of Healthy Overweight Adults. Sci. Rep..

[48] Irie J., Inagaki E., Fujita M., Nakaya H., Mitsuishi M., Yamaguchi S., Yamashita K., Shigaki S., Ono T., Yukioka H. (2020). Effect of oral administration of nicotinamide mononucleotide on clinical parameters and nicotinamide metabolite levels in healthy Japanese men. Endocr. J..

[49] Stevens M.J., Li F., Drel V.R., Abatan O.I., Kim H., Burnett D., Larkin D., Obrosova I.G. (2007). Nicotinamide reverses neurological and neurovascular deficits in streptozotocin diabetic rats. J. Pharmacol. Exp. Ther..

[50] Knip M., Douek I.F., Moore W.P., Gillmor H.A., McLean A.E., Bingley P.J., Gale E.A., European Nicotinamide Diabetes Intervention Trial Group (2000). Safety of high-dose nicotinamide: A review. Diabetologia.

[51] Williams A., Ramsden D. (2005). Nicotinamide: A double edged sword. Parkinsonism Relat. Disord..

[52] Zhang J.G., Zhao G., Qin Q., Wang B., Liu L., Liu Y., Deng S.C., Tian K., Wang C.Y. (2013). Nicotinamide prohibits proliferation and enhances chemosensitivity of pancreatic cancer cells through deregulating SIRT1 and Ras/Akt pathways. Pancreatology.

[53] Peled T., Shoham H., Aschengrau D., Yackoubov D., Frei G., Rosenheimer G.N., Lerrer B., Cohen H.Y., Nagler A., Fibach E. (2012). Nicotinamide, a SIRT1 inhibitor, inhibits differentiation and facilitates expansion of hematopoietic progenitor cells with enhanced bone marrow homing and engraftment. Exp. Hematol..

[54] Kraus D., Yang Q., Kong D., Banks A.S., Zhang L., Rodgers J.T., Pirinen E., Pulinilkunnil T.C., Gong F., Wang Y.C. (2014). Nicotinamide N-methyltransferase knockdown protects against diet-induced obesity. Nature.

[55] Trammell S.A., Brenner C. (2015). NNMT: A Bad Actor in Fat Makes Good in Liver. Cell Metab..

[56] Ruf S., Hallur M.S., Anchan N.K., Swamy I.N., Murugesan K.R., Sarkar S., Narasimhulu L.K., Putta V., Shaik S., Chandrasekar D.V. (2018). Novel nicotinamide analog as inhibitor of nicotinamide N-methyltransferase. Bioorg. Med. Chem. Lett..

[57] Marshall A., Alam U., Themistocleous A., Calcutt N., Marshall A. (2021). Novel and Emerging Electrophysiological Biomarkers of Diabetic Neuropathy and Painful Diabetic Neuropathy. Clin. Ther..

[58] Drexel H. (2007). Nicotinic acid in the treatment of hyperlipidaemia. Fundam. Clin. Pharmacol..

[59] Purushotham A., Schug T.T., Xu Q., Surapureddi S., Guo X., Li X. (2009). Hepatocyte-specific deletion of SIRT1 alters fatty acid metabolism and results in hepatic steatosis and inflammation. Cell Metab..

[60] Imamura H., Nagayama D., Ishihara N., Tanaka S., Watanabe R., Watanabe Y., Sato Y., Yamaguchi T., Ban N., Kawana H. (2017). Resveratrol attenuates triglyceride accumulation associated with upregulation of Sirt1 and lipoprotein lipase in 3T3-L1 adipocytes. Mol. Genet. Metab. Rep..

[61] Fernyhough P., Calcutt N.A. (2016). New Directions in Diabetic Neuropathy: Evolution or Extinction?. Int. Rev. Neurobiol..

[62] Benyo Z., Gille A., Kero J., Csiky M., Suchankova M.C., Nusing R.M., Moers A., Pfeffer K., Offermanns S. (2005). GPR109A (PUMA-G/HM74A) mediates nicotinic acid-induced flushing. J. Clin. Investig..

[63] Bogan K.L., Brenner C. (2008). Nicotinic acid, nicotinamide, and nicotinamide riboside: A molecular evaluation of NAD+ precursor vitamins in human nutrition. Annu. Rev. Nutr..

[64] Tunaru S., Kero J., Schaub A., Wufka C., Blaukat A., Pfeffer K., Offermanns S. (2003). PUMA-G and HM74 are receptors for nicotinic acid and mediate its anti-lipolytic effect. Nat. Med..

[65] Diguet N., Trammell S.A.J., Tannous C., Deloux R., Piquereau J., Mougenot N., Gouge A., Gressette M., Manoury B., Blanc J. (2018). Nicotinamide Riboside Preserves Cardiac Function in a Mouse Model of Dilated Cardiomyopathy. Circulation.

[66] Houtkooper R.H., Canto C., Wanders R.J., Auwerx J. (2010). The secret life of NAD+: An old metabolite controlling new metabolic signaling pathways. Endocr. Rev..

[67] Sauve A.A., Wolberger C., Schramm V.L., Boeke J.D. (2006). The biochemistry of sirtuins. Annu. Rev. Biochem..

[68] Roy Chowdhury S.K., Smith D.R., Saleh A., Schapansky J., Marquez A., Gomes S., Akude E., Morrow D., Calcutt N.A., Fernyhough P. (2012). Impaired adenosine monophosphate-activated protein kinase signalling in dorsal root ganglia neurons is linked to mitochondrial dysfunction and peripheral neuropathy in diabetes. Brain.

[69] Sadi G., Konat D. (2016). Resveratrol regulates oxidative biomarkers and antioxidant enzymes in the brain of streptozotocin-induced diabetic rats. Pharm. Biol..

[70] Zhou Y., Lian S., Zhang J., Lin D., Huang C., Liu L., Chen Z. (2018). Mitochondrial Perturbation Contributing to Cognitive Decline in Streptozotocin-Induced Type 1 Diabetic Rats. Cell Physiol. Biochem..

[71] Xu J., Jackson C.W., Khoury N., Escobar I., Perez-Pinzon M.A. (2018). Brain SIRT1 Mediates Metabolic Homeostasis and Neuroprotection. Front. Endocrinol..

[72] Xu L., Xu S., Lin L., Gu X., Fu C., Fang Y., Li X., Wang X. (2018). High-fat Diet Mediates Anxiolytic-like Behaviors in a Time-dependent Manner Through the Regulation of SIRT1 in the Brain. Neuroscience.

[73] Biessels G.J., Bril V., Calcutt N.A., Cameron N.E., Cotter M.A., Dobrowsky R., Feldman E.L., Fernyhough P., Jakobsen J., Malik R.A. (2014). Phenotyping animal models of diabetic neuropathy: A consensus statement of the diabetic neuropathy study group of the EASD (Neurodiab). J. Peripher. Nerv. Syst..

[74] Choi J., Chandrasekaran K., Demarest T.G., Kristian T., Xu S., Vijaykumar K., Dsouza K.G., Qi N.R., Yarowsky P.J., Gallipoli R. (2014). Brain diabetic neurodegeneration segregates with low intrinsic aerobic capacity. Ann. Clin. Transl. Neurol..

[75] Chandrasekaran K., Muragundla A., Demarest T.G., Choi J., Sagi A.R., Najimi N., Kumar P., Singh A., Ho C.Y., Fiskum G. (2017). mGluR2/3 activation of the SIRT1 axis preserves mitochondrial function in diabetic neuropathy. Ann. Clin. Transl. Neurol..

[76] Lauria G., Hsieh S.T., Johansson O., Kennedy W.R., Leger J.M., Mellgren S.I., Nolano M., Merkies I.S., Polydefkis M., Smith A.G. (2010). European Federation of Neurological Societies/Peripheral Nerve Society Guideline on the use of skin biopsy in the diagnosis of small fiber neuropathy. Report of a joint task force of the European Federation of Neurological Societies and the Peripheral Nerve Society. Eur. J. Neurol..

[77] Lauria G., Lombardi R., Borgna M., Penza P., Bianchi R., Savino C., Canta A., Nicolini G., Marmiroli P., Cavaletti G. (2005). Intraepidermal nerve fiber density in rat foot pad: Neuropathologic-neurophysiologic correlation. J. Peripher. Nerv. Syst..

[78] Chandrasekaran K., Anjaneyulu M., Inoue T., Choi J., Sagi A.R., Chen C., Ide T., Russell J.W. (2015). Mitochondrial transcription factor A regulation of mitochondrial degeneration in experimental diabetic neuropathy. Am. J. Physiol. Endocrinol. Metab..

[79] Liang X., Yang L., Qin A.R., Ly J., Liederer B.M., Messick K., Ma S., Zak M., Dragovich P.S., Dean B.J. (2014). Measuring NAD(+) levels in mouse blood and tissue samples via a surrogate matrix approach using LC-MS/MS. Bioanalysis.

[80] Russell J.W., Berent-Spillson A., Vincent A.M., Freimann C.L., Sullivan K.A., Feldman E.L. (2008). Oxidative injury and neuropathy in diabetes and impaired glucose tolerance. Neurobiol. Dis..

