# Supplementary material for: NAD+ Precursors Repair Mitochondrial Function in Diabetes and Prevent Experimental Diabetic Neuropathy
Source: Int J Mol Sci. 2022 Apr 28;23(9):4887. doi: 10.3390/ijms23094887 (PMC9102948; doi:10.3390/ijms23094887)
Supplement: Supplementary file 1 [file ijms-23-04887-s001.zip › ijms-1664997-supplementary.pdf]

Supplementary Data

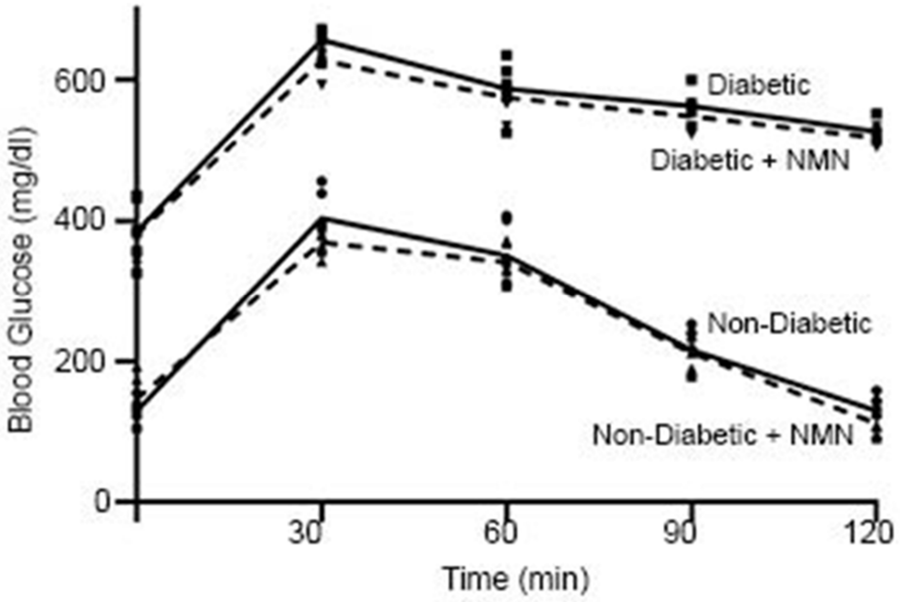

Figure S1. Intraperitoneal glucose tolerance test was done as described in Ref 11. Statistical data for area under the curve for the glucose tolerance test is showed significant differences between non-diabetic and STZ diabetic mice (Area Under the Curve = mg*min/dL X 10^3); non-diabetic = 3.6 ± 0.3 vs. STZ mice 9.1 ± 10 but not between STZ and STZ+NMN (8.9 ± 0.8).

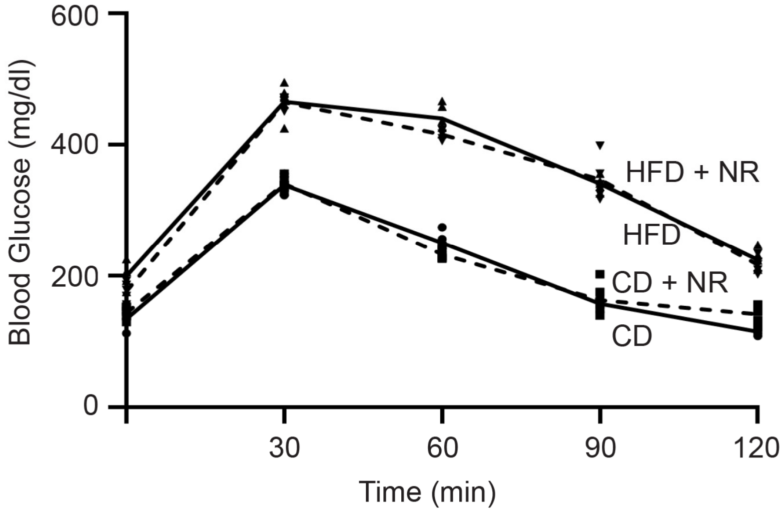

Figure S2. The intraperitoneal glucose tolerance test was conducted as described in [11]. Statistical data for area under the curve for the glucose tolerance test showed significant differences between CD- and HFD-fed mice, but there was no significant difference in the AUC between HFD and HFD + NR mice. The statistical significance is shown in Table 3.
